# Prevalence of Chronic Pain, Treatments, Perception, and Interference on Life Activities: Brazilian Population-Based Survey

**DOI:** 10.1155/2017/4643830

**Published:** 2017-09-26

**Authors:** Juliana Barcellos de Souza, Eduardo Grossmann, Dirce Maria Navas Perissinotti, Jose Oswaldo de Oliveira Junior, Paulo Renato Barreiros da Fonseca, Irimar de Paula Posso

**Affiliations:** ^1^Universidade Federal de Santa Catarina, Hospital Universitário, Florianópolis, SC, Brazil; ^2^Sociedade Brasileira para o Estudo da Dor, São Paulo, SP, Brazil; ^3^Universidade Federal do Rio Grande do Sul, Porto Alegre, RS, Brazil; ^4^Equipe de Controle da Dor Disciplina de Anestesiologia Faculdade de Medicina da Universidade de São Paulo, Laboratório Sujeito e Corpo (SUCOR) do Instituto de Psicologia da USP, São Paulo, SP, Brazil; ^5^Escola de Cancerologia Celestino Bourroul da Fundação Antônio Prudente de São Paulo, São Paulo, SP, Brazil; ^6^Serviço de Anestesiologia e Clinica de Dor Oncológica do GRUPO COI, Rio de Janeiro, RJ, Brazil; ^7^Faculdade de Medicina do ABC, Centro de Treinamento de Anestesiologia, São Paulo, SP, Brazil

## Abstract

**Background and Objectives:**

Chronic pain affects between 30% and 50% of the world population. Our objective was to estimate the prevalence of chronic pain in Brazil, describe and compare differences between pain types and characteristics, and identify the types of therapies adopted and the impact of pain on daily life.

**Methods:**

Cross-sectional study of a population-based survey with randomized sample from a private database. The interviews were conducted by phone. 78% of the respondents aged 18 years or more agreed to be interviewed, for a total of 723 respondents distributed throughout the country. Independent variables were demographic data, pain and treatment characteristics, and impact of pain on daily life. Comparative and associative statistical analyses were conducted to select variables for nonhierarchical logistic regression.

**Results:**

Chronic pain prevalence was 39% and mean age was 41 years with predominance of females (56%). We found higher prevalence of chronic pain in the Southern and Southeastern regions. Pain treatment was not specific to gender. Dissatisfaction with chronic pain management was reported by 49% of participants.

**Conclusion:**

39% of interviewed participants reported chronic pain, with prevalence of females. Gender-associated differences were found in intensity perception and interference of pain on daily life activities.

## 1. Introduction

Chronic pain is a multidimensional health condition defined by the International Association for the Study of Pain (IASP) as pain persisting for more than six months [1], although being much more related to peripheral and central nervous system sensitization than to exclusive duration time. Currently, chronic pain is no longer considered just a symptom, but rather a disease, classified as R 52.1 under the wrong name of intractable disease, in the tenth edition of the International Code of Diseases (ICD10). There are some controversial of chronic pain definition as a “medical unexplained pain” supporting pain as a symptom or as a degree of depression, irritability, and anxiety [2], in contrast to chronic pain as a disease or injury related to long-lasting changes on peripheral and central neural responses resulting in sensitization [3].

Chronic pain affects one-third to fifty percent of the population [4]. A multicenter study carried out in 1998 by the World Health Organization (WHO) has shown prevalence of chronic pain in 22% of the world population; however Brazilian data collected in Rio de Janeiro have a shown prevalence of 31% [5]. In Brazil, other population-based studies have mapped the prevalence of chronic pain in some urban regions such as São Paulo [6] with 29%, Florianópolis [7] with 26%, and Salvador [8] with 40%.

Most epidemiologic studies show higher prevalence among females, the same being true for advanced age [7–9]. It is estimated that the incidence of pain among adults between the ages of 18 and 25 years is approximately 14% versus 62% among those above 75 years [4]. Other large demographic studies reported at this meta-analysis [4] have found that adults between 18 and 39 years may have prevalence rates above 30% [6], and increased prevalence seems to be associated with musculoskeletal system aging.

Chronic pain is considered a health crisis due to its high prevalence and associated physical and emotional incapacity. The socioeconomic impact of lumbar and cervical pain is among the 10 major causes of incapacity by the world classification of disabilities [10]. It is estimated that chronic pain is one of the major causes of disability in many regions of the world, in developed or developing countries, and may inhibit people's ability to carry out labor and daily activities, in addition to impairing their mobility [9].

Musculoskeletal disorders and osteoarthritis are among the 20 major causes of incapacity worldwide [4]. In Brazil, the five major causes of incapacity by years lived are low back pain, followed by severe depressive disorder, anxiety disorder, throat pain, and asthma [4]. The economic burden related to chronic pain and its associated level of disability supports the development of evidence-based health policies adapted to epidemiologic, socioeconomic, and cultural realities of each region.

Brazil is a country with continental dimensions, with more than 8.5 million square kilometers, being the fifth most populous country of the world, characterized by significant space distribution heterogeneity, with high geographic density in the Southeastern region and owner distribution in the Northern region. It is politically, geographically, and administratively divided into 26 states and one Federal District. Regional division in five major regions is based on the interrelation of natural, economic, historic, and cultural aspects.

The five major regions are Northern, Northeastern, Midwest, Southeastern, and Southern [11]. There are deep inter- and intraregional differences because as per data of the Brazilian Institute of Geography and Statistic (IBGE) only three among 26 states and the Federal District are responsible for more than half the national Gross Domestic Product (GDP), and São Paulo predominates with 11% of GDP production [11].

The heterogeneity of the country is translated into life expectation, income, and education level, data which make up the human development index (HDI) calculation. In 2010, precarious HDI of the Northeastern region below 0.6 contrasts with Southern and Southeastern regions with high HDI above 0.82 [12]. Besides these differences, the history of each Brazilian region was very different, as their customs, religion, and beliefs.

Epidemiologic and associative studies have shown the prevalence of pain as a function of socioeconomic and education levels [7, 8]. The variability of human and material resources among Brazilian regions justifies the need for population-based studies investigating a representative sample of Brazilians from each state of the country. Public health policy planning depends on data collection studies highlighting the differences among Brazilian regions and associated demographics in terms of lifestyle and patient preferences for chronic pain management based on cultural beliefs.

The major objective of this population-based study was to estimate the prevalence of chronic pain in Brazil and in its five major regions. Some differences of chronic pain between gender were assessed by analyses of some independent variables of pain.

## 2. Methods

**Figure 1:**
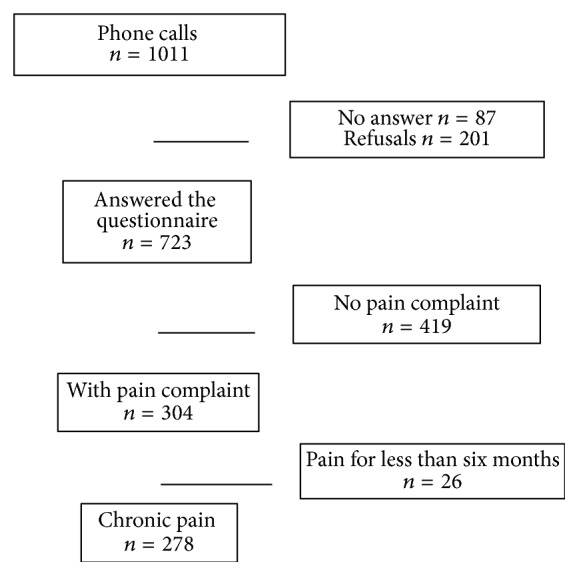
Study design flowchart.

This was an observational study, cross-sectional population-based survey with 1011 participants aged 18 years or above. Our first sample size was determined at 1000 and extended to 1011 people in order to cover the demographic density of the population of each state and of the Federal District (FD), like proposed by IBGE [11]. Probabilistic sample was defined as function of demographic density of the population of each state and FD, and weight of variables was higher on populated state. Sample was randomized from the private database CEO Marketing with 1 million Brazilian cell phone numbers. According to IBGE data, in 2013, 73% of Brazilians have a cell phone, and in 2004 52,5% of rural population has a cell phone in contrast to 82,3% of urban population. In 2017, the density was 116,65 cell phones/100 people [11]. In case of inexistent or inactive cell number, the next number of the list was included until the call was completed. After three attempts in different times and days, 87 phone calls were not answered.  Figure 1 shows study design flowchart.

Data were collected by a trained interviewer, from November 2015 to February 2016. Interviews lasted from 3 to 20 minutes by participant, according to answers to the questionnaire. Data collection tool was proposed by the* “Federación Latino-Americana para el Estudo del Dolor,”* to investigate pain prevalence in Latin America, not published.

Structured questions to access the outcome variable of chronic pain were: “Do you currently feel any type of pain or are you currently taking any painkiller?” followed by “For how long do you feel this pain? Years, months or days” [13], validated in Brazilian-Portuguese [14]. Chronic pain was defined by responders to live with persistent during the last six or more months.

Pain characterization variables were as follows: (a) cause or diagnosis was self-reported; (b) weekly frequency of chronic pain was assessed by answering as pain was less than 1 day/week, 1 or 2 days/week, 3-4 days/week, or 5 or more days/week; (c) pain crises duration was assessed by answers options as momentary, few hours, one day, and constant [14–18]; (d) pain intensity was evaluated by the Numerical Rating Scale to rate the pain intensity from 0 to 10 [14–18]; (e) pain location was assessed through a body map template [15], each body part was read, and participants should respond if they were painful or not; (f) impact of pain on daily life activities was assessed by NRS from 0 to 10 and a Likert-type scale of “not at all a problem,” “minor problem,” “moderate problem,” or “serious problem” [15] for assessing pain interference with self-care, walking, work, social life, irritants and emotional effects, sadness or depression, sexual life, and sleep, validated in Brazilian-Portuguese [14, 18].

Pain characterization continuous variables were as follows: (a) pain intensity was evaluated by the Numerical Rating Scale to rate the pain intensity from 0 to 10 [14, 16] and (b) impact of pain on daily life activities was also assessed by a scale from 0 to 10 [14, 16] and validated in Brazilian-Portuguese [14, 18].

Independent variables were divided into three blocks: sociodemographic, pain characterization, and treatment characterization. Variables were self-reported by respondents. Sociodemographic variables were age in years and gender (female or male). State and region were acquired by the area code, which is the prefix of telephones for contacting each participant.

Treatment characterization variables were medical specialty of professionals treating respondents' chronic pain, drugs used to manage pain, other therapies received in addition to drug, and pain management self-evaluation.

### 2.1. Statistical Analysis

Data analysis was descriptive with mean and 95% confidence interval, with estimates of relative frequency for chronic pain prevalence by Brazilian region and gender. Although data collection was proportional to Brazilian states population, we decided to group data by geopolitical region to improve statistical power, that is, less freedom for interferences. Linear association Chi-square test (*p* < 0.05) was used for bivariate analyses to check the prevalence and raw association between genders and independent variables characteristic of pain and treatment. Data with association to gender and significance below 0.10 were included in nonhierarchical logistic regression analysis, Wald advance method, and criterion to remain in the *p* < 0.05 model. SPSS version 20.0 for Windows was used for data analysis.

## 3. Results

This population-based study has interviewed 723 people distributed through all Brazilian states, with a 78% participation rate. Mean age of participants was 38 years with slight female predominance (52%) and 91% of responders were adults, aged between 20 and 59 years. We have selected 1011 cell phones but only 723 adults between 18 and 75 years have answered in all Brazilian states and in the Federal District. After three attempts in different times and days, 87 phone calls were not completed. Refusal to answer the questionnaire has varied from 49% in the Southern region to 10% in the Southeastern region and was statistically significant by association of Chi-square test (*p* < 0.0001), interfering with study sample homogeneity, with less representation of Southern, Midwest, Western, and Northern regions.

Female participants' answers were slightly predominant in Brazilian regions, except for the Southern region where males have predominated; however there has been no significant association between gender and Brazilian region (Pearson Chi-square 0.444; *p* = 0.98). Sample description by region in age and gender is shown in Table 1.

Among females, 304 respondents (56.6%) reported having pain or being under pharmacological treatment for pain control. Pain self-report was present in 42% of the sample (*n* = 723), percentage which has decreased to 30% when accounting for the 87 respondents that did not answer this item. When phone contacts for the interview failed, these were excluded from subsequent statistical analysis.

Pain prevalence was significantly different among Brazilian regions: 25% in Midwest region; 32% in Northeastern region; 42% in Northern region; 44% in Southeastern region; and 47% in Southern region.

Pain prevalence according to gender was also significantly different among Brazilian regions: in Midwest region, it was 50% for males and females; in Northeastern region, it was 55% for females and 45% for males; in Northern region it was 57% for females and 43% for males; in Southeastern region it was 56% for females and 44% for males; and in Southern region it was 61% for females and 39% for males (Table 2).

### 3.1. Pain Characterization

Mean age of respondents reporting chronic pain was not different between genders; however, pain intensity and interference with daily activities were significantly higher among females as compared to males (Table 3). Pain crises frequency and duration were significantly higher among females, who have also reported further interference of pain in self-care, work, sexual life, and sleep interruption (Table 3).

**Figure 2:**
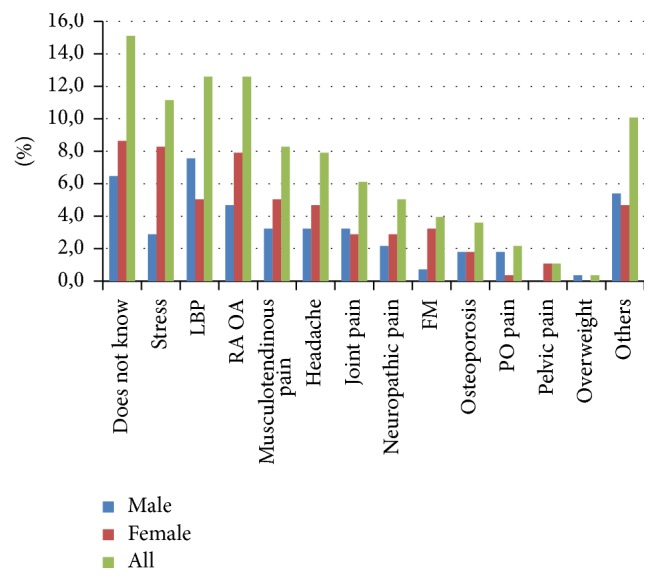
Percentage of cause and diagnosis associated with chronic pain with regard to gender. In Brazil in 2015-2016. LBP: low back pain, RA OA: rheumatoid arthritis or osteoarthritis, FM: fibromyalgia, and PO pain: postoperative pain.

**Figure 3:**
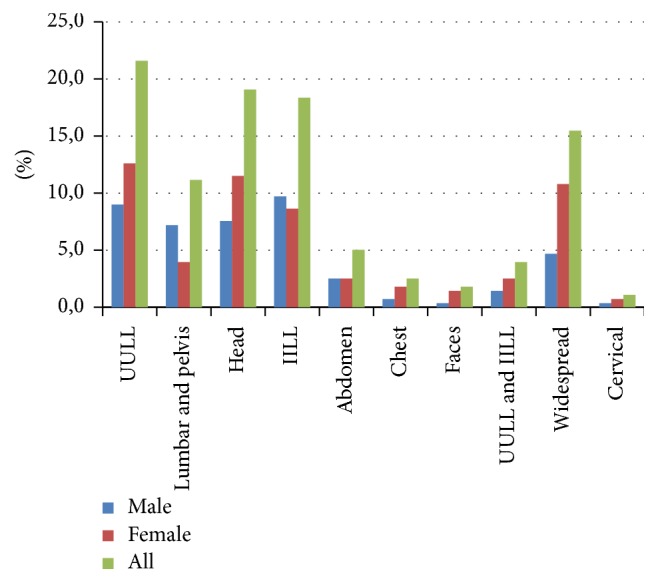
Location of pain reported by female and male participants. In Brazil in 2015-2016. UULL: upper limbs and IILL: lower limbs.

Cause of pain was unknown by 15% of respondents. Low back pain and/or sacroiliac region problems were mentioned as the cause or diagnosis by 13% of respondents. Rheumatic diseases such as osteoarthritis and arthritis were also mentioned by 13% of respondents (Figure 2). In regard to pain location, chronic pain was predominant in upper limbs (22%), head and neck (19%), and lower limbs (18%), followed by widespread pain (15%) (Figure 3). Pain location and cause were not significantly associated with gender.

Pain-induced disability was reported by 52.7% of participants, significantly associated with females, with 65.3% versus 34.7% of males who reported pain-associated incapacity (Chi-square 9.71; *p* < 0.01). Additionally, 87.6% of respondents have described disability duration for less than six months.

### 3.2. Pain Management Characterization

**Figure 4:**
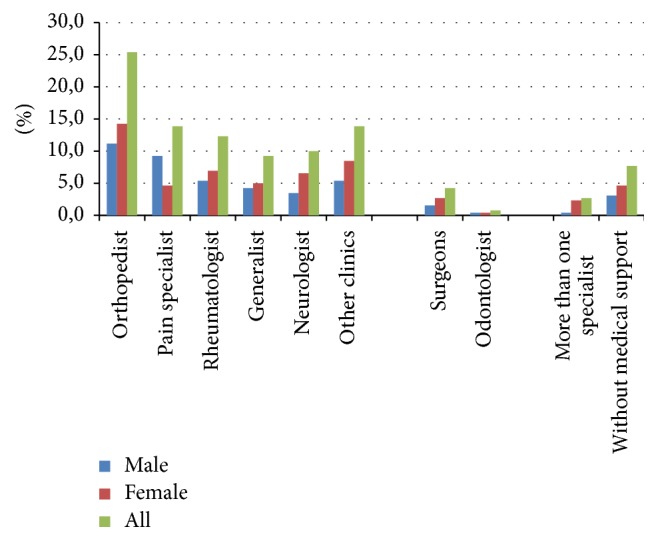
Medical specialties chosen as first option of Brazilians with chronic pain as a function of gender 2015-2016.

The demand for a medical specialty to manage chronic pain was not different among genders. Orthopedists (25%) were the most often looked for physicians to manage chronic pain, followed by pain specialists (14%) and clinicians, such as rheumatologists (12%), neurologists (10%), and general practitioners (9%). Approximately 8% of respondents have reported not having medical follow-up for the management of their pain.  Figure 4 shows frequency of consultations by medical specialty.

Among proposed therapies, 75% of participants with chronic pain used drugs; however 12% have reported not looking for pain management. Alternative therapies were the option for 2% of respondents and blockades were also an option for 2% of respondents, while 9% respond with “other.” There has been no significant difference among genders in the choice of pain management therapies (Chi-square = 0.367; *p* = 0.544) or among Brazilian regions (Chi-square = 3.713; *p* = 0.004). Among pharmacological options, there was a predominance of anti-inflammatory analgesics diclofenac, naprofen, aspirin, ibuprofen, and ketorolac, which were the therapeutic choice for 3.2% (*n* = 67) of respondents. This was followed by nonopioid analgesics dipyrone or acetaminophen, chosen as therapy by 22.2% of respondents (*n* = 28). Antidepressants and opioids were reported by 12.7% (*n* = 16) and 10.3% (*n* = 13), respectively, and anticonvulsants were the therapeutic option for 1.6% (*n* = 2) of respondents.

Self-medication was not predominant in the sample; one-fifth have reported self-medication for pain management (19.1%), as opposed to 68.3% looking for medical assistance for drug prescription and 4.7% making use of pharmacists. Three percent have resorted to family or advertising or to Indians for drug prescription.

Most respondents make use of nonpharmacological therapies such as homemade drugs (23%, *n* = 56), physiotherapy (9.4%, *n* = 23), relaxation (7.8%, *n* = 19), acupuncture (6.1%, *n* = 15), alternative medicine (2.0%, *n* = 5), transcutaneous electric nerve stimulation (TENS) (1.2%, *n* = 3), or other nonspecified conservative treatments which were the option for 50.4% (*n* = 123).

The effect of pain treatments was self-evaluated by 48.7% of responders as “no effect” or “minor effect,” and 36.4% classified it as “good effect” in contrast to just 14.9% who classified the effect of their treatment for chronic pain as “very good” or “excellent.”

### 3.3. Logistic Regression

Variables with association between genders, previously defined by bivariate analysis, were included in the logistic regression. Gender-associated factors in those with chronic pain and remaining in the model were pain intensity (OR 1.17, CI 95% 1.02–1.34, *p* < 0.05) and interference (OR 1.20, CI 95% 1.04–1.37, *p* < 0.05) with daily life activities, with higher association in intensity with the female gender, and with *R*^2^ of 0.115. Both continuous variables and no categorical variable have remained in the final model (Table 4).

## 4. Discussion

Chronic pain prevalence in the Brazilian population was 39%, with mean age of 41 years and female predominance of 56%. Chronic pain prevalence was higher in Southern and Southeastern regions, with 43% and 40%, respectively. Participants with chronic pain in these two regions represented 84% of total chronic pain patients of the Brazilian sample. This prevalence is high as compared to WHO expectations; however it is equivalent to that of developed countries with high prevalence of chronic pain, as shown by a postal survey which has evidenced 31.7% prevalence of chronic pain in the French population and a telephone survey carried out in the United Kingdom, with percentage of corrected answers of 52%, evidencing chronic pain prevalence in 48% [19], and a different survey carried out in England where the prevalence has reached 50% [4].

Midwest and Northeastern regions had the lowest chronic pain prevalence rates, 24% and 30%, respectively, data equivalent to worldwide prevalence shown by a WHO multicenter study [5], although a study carried out in Goiânia, city of the Midwest region, has shown a prevalence of 52% in healthy elderly people [20].

Brazilian Northern and Northeastern regions are classified by HDI as less favored regions with regard to life expectation, education level, and per capita income, as compared to Southern and Southeastern regions; despite this, Northern and Northeastern regions have lower pain prevalence. In a way, these data are conflicting with epidemiologic results of the association of chronic pain and socioeconomic factors, where low income and low education level are risk factors. Data of a USA survey from 2009 to 2010 point to the association between chronic low back pain and low education and income levels, even when adjusted to age and comorbidities [21].

However, in addition to socioeconomic level and HDI, social inequality and difficult access to specialized health services [22], age [23], and gender [24] may influence the access to diagnosis and pain and comorbidities treatment. The association of other ecologic socioeconomic factors allows suggesting that public health policies planning be adapted to such conditions and to the heterogeneous characteristics of Brazilian regions.

Chronic pain location is varied and is associated with different causes or clinical diagnoses, such as musculoskeletal, connective tissue, and nervous system diseases, injuries, or traumas. The prevalence of chronic pain was evaluated by a systematic review and meta-analysis including data of pain prevalence in three different populations as general, elderly, and workers [25]. This study found that migraine is the complaint of 42% of general population, 30% of the elderly, and 51% of workers; and daily chronic headache has a prevalence of 5% in general population and 10% of workers [25]. In this study, 19% of respondents have reported head and neck pain; however just 7.9% have reported headache. Fifteen percent of respondents did not know the reason for the pain, values which are lower than that of previous studies where 34% of general population report nonspecific chronic pain and 62% of the elderly report nonspecific chronic pains [25].

Joint pain was mentioned by 6.1% and arthritis and osteoarthritis were mentioned by 12.6%; however, previous studies have evidenced that 14% of general population and 34% of the elderly have reported joint pain [25]. Low back pain affected 12.6% of respondents, which is lower than previous studies evidencing mean prevalence of 21% in the general population, 28% in the elderly, and 52% among workers. The prevalence of musculoskeletal pain was only 8.3% among Brazilians from our sample, versus 25%, on adults from general population [25]; 44% of elderly people [25]; and 79% among workers [25].

Widespread chronic pain was reported by 15% of participants, which is higher than data of other studies where the incidence in the general population was 7% and in the elderly 19% [25].

Fibromyalgia was reported as a pain diagnosis by 1.4% of respondents, rates which are lower than 2.5% evidenced in a study carried out in the state of São Paulo [26] and 6% evidenced by meta-analysis and systematic review [4]. Differences in prevalence may be explained by the difficult access to accurate diagnosis [22]; also they may be maximized by risk situations and physical or emotional stress, such as results of studies showing increased prevalence of fibromyalgia and posttraumatic stress in the city of New York after the terrorist attacks [27]. Multiple studies identified comorbidity of posttraumatic stress disorder and chronic pain [28, 29] and other psychiatric disorders [2]. The stigmatization of people suffering from chronic pain should be minimized by psychiatric and psychotherapy treatment.

Chronic pain management in Brazil was not different between males and females. Both genders primarily resort to orthopedics, rheumatology, and neurology specialties to control their pain. The detection of potentially hazardous anti-inflammatory analgesics among pharmacological options is an alert for the need of qualitative improvement of assistance to chronic pain patients. Previous studies reported that about 20% of patients take pain medication without a doctor advice [30] that can aggravate other problems as increasing gastric and cardiovascular risks [31]. The Brazilian health system allows some patients to have direct access to specialists without necessarily being referred by a family physician or generalist, as opposed to other developed countries [22, 25], the population of which is object of study of most epidemiologic data available in the scientific literature. In Brazil, access to anti-inflammatory is easy because it does not require a medical prescription and is also available on the pharmacy shelf. In contrast, analgesic opioids access was very difficult and required a difficult process with specific medical prescription.

There are three basic ways to access health services in Brazil: public, private, and health insurance. The type of health service choice might be described as a confusion variable for the access to diagnosis, specialized services, and patients' management. This variable, with high confusion potential, was not investigated in this study. There are differences between the population using public or private health insurance; however data of a North American study state that patients with chronic low back pain resort three times more to private or governmental health insurance reimbursements, in addition to more frequent medical visits of 3.35 for ≥10 health consultations in the last year, as compared to those without chronic low back pain [21].

Almost 50% of our sample classified the effect of our chronic pain treatment ineffective on pain management. The objectives of IASP and of the Brazilian Society for the Study of Pain (SBED) include promoting the awareness of the need for accurate diagnosis, adequate management, and the importance of health professionals' qualification to assist people with chronic pain. Considering that 80% of all medical consultations are motivated by pain and that 30% to 40% of the population have associated chronic pain or as the reason for consultation, health professionals should be qualified and updated to diagnose and treat this public health problem associated with high national and worldwide health expenditures.

A challenge for chronic pain management is the multiprofessional approach. Several studies show the medium and long term success of multifactorial health approaches; however they stress limitations in human and structural resources [32].

Our study has some methodological limitations related to information bias and sample size, because participants were selected based on a private cell phones database, limiting the access just to those using this communication tool, in addition to the rate of refusal to answer the questionnaire, as well as missing data due to failed telephone contact. There has been significant difference in participation by Brazilian region, being the Southeastern region with lower refusal rate as opposed to the Southern region with the highest refusal rate, especially by female participants. Another important limitation was that the sample-associated regions were defined by the area code used to select participants; however dwelling region of respondents was not confirmed. Telephone numbers of area codes of capitals were randomly selected; however in some more populous states, such as São Paulo, the code refers only to the capital of the state of São Paulo, while in Santa Catarina, for example, capital code also comprises the South coast of the state or even Rio Grande do Sul where the code refers to the whole state. Probably the prevalence of pain in Brazil was underestimated because elderly population were underrepresented on our sample. According to IBGE data 14% of Brazilian population are 60 years old or more, in contrast to our 5% (*n* = 37) of responders; among elderly people 62% (*n* = 23) reported chronic pain. Future studies should assess some confounding variables as socioeconomic status, educational level, health service (public or private), psychiatric comorbidities, and where the responder lives (as urban, rural, or village).

Our study has as a positive point the national coverage, based on interviews of Brazilians in all Brazilian states and regions. These is the first population-based study that accesses chronic pain prevalence on Brazil. Our data provide a better understanding of the impact of chronic pain on Brazilian population and could contribute to improving planning and to allowing the optimization of strategies and the enhancement of public campaigns and policies aimed at the adequate management of chronic pain.

## 5. Conclusion

Our study shows that in Brazil chronic pain affects almost 40% of adults and the elderly, with predominance of females. Gender-associated differences were found in intensity perception and pain interference with daily life activities. Other symptoms, perceptions, and profile of using health services were not different between genders. Most people resort to specialists in the areas of orthopedics, rheumatology, and neurology for pain management, and the most widely used/prescribed drugs for chronic pain were anti-inflammatory analgesics.

## Figures and Tables

**Table 1:** Sample characterization by Brazilian region.

	Contacts	Respondents	Age in years	Female gender
		*n* (%)	Mean (CI 95%)	*n* (%)
Northern	59	33 (56)	36.8 (32.6–41.0)	17 (52)
Northeastern	138	96 (70)	36.3 (33.9–38.7)	49 (51)
Midwest	32	17 (53)	37.8 (28.1–47.6)	9 (56)
Southeastern	577	517 (90)	38.2 (37.1–39.3)	269 (52)
Southern	118	60 (51)	39.0 (35.6–42.4)	29 (48)

Total	924	723 (78)	37.9 (37.0–38.9)	373 (52)

**Table 2:** Chronic pain prevalence by gender and human development index by region.

	Chronic pain prevalence (*n* = 278) % in the region (% in Brazil)	Prevalence among females % in the region (% in Brazil)	HDI 2010 Max–Min by region^*∗*^
Northern	36% (5)	67% (5)	0.708–0.646
Northeastern	30% (10)	52% (10)	0.684–0.631
Midwest	24% (1)	50% (1)	0.824–0.725
Southeastern	40% (75)	57% (75)	0.783–0.731
Southern	43% (9)	58% (10)	0.774–0.746

Total	39% (100)	57% (100)	

^*∗*^http://www.atlasbrasil.org.br/2013/pt/ranking/.

**Table 3:** Comparison of pain characteristics between genders in the Brazilian population 2015-2016.

	Male	Female	Total	Statistical analysis
Age				
Mean (CI 95%)	42.2 (39.7–44.7)	40.5 (38.7–42.3)	41.2 (39.7–42.7)	NS

Intensity				
(0 to 10)	6.0 (5.6–6.4)	7.0 (6.6–7.3)	6.5 (6.3–6.8)	*t* = −3.43 *p* < 0.01

Weekly frequency				Chi-square 4.16
				*p* < 0.05
Less than 1	38.8% (47)	31.2% (49)	34.5% (96)	
1-2 days	28.9% (35)	21.7% (34)	24.8% (69)	
3-4 days	5.0% (6)	9.6% (15)	7.6% (21)	
5–7 days	27.3% (33)	37.6% (59)	33.1% (92)	

Crises duration				Chi-square 6.26
				*p* < 0.05
Momentary	15.8% (19)	12.8% (20)	14.1% (39)	
Few hours	28.3% (34)	17.9% (28)	22.5% (62)	
One day	26.7% (32)	23.7% (37)	25.9% (69)	
Constant	29.2% (35)	45.5% (71)	38.4% (106)	

Interference with DLAs				
(0–10)	6.3 (5.8–6.7)	7.3 (6.9–7.6)	6.8 (6.54–7.1)	*T* = 3.71 *p* < 0.01

Interference with self-care				Chi-square 9.9
				*p* < 0.01
Not at all a problem	46.8% (51)	27.6% (37)	36.2% (88)	
Minor problem	33.0% (36)	39.6% (53)	36.6% (89)	
Moderate problem	11.9% (13)	14.9% (20)	13.6% (33)	
Serious problem	8.3% (9)	17.9% (24)	13.6% (33)	

Interferes with walking				NS
Not at all a problem	37.7% (43)	37.7% (55)	37.7% (98)	
Minor problem	27.2% (31)	24.0% (35)	25.4% (66)	
Moderate problem	17.5% (20)	18.5% (27)	18.1% (47)	
Serious problem	17.5% (20)	19.9% (29)	18.8% (49)	

Interferes with work				Chi-square 3.96
				*p* < 0.05
Not at all a problem	21.9% (25)	20.3% (30)	21.0% (55)	
Minor problem	33.3% (38)	25.0% (37)	28.6% (75)	
Moderate problem	29.8% (34)	25.0% (37)	27.1% (71)	
Serious problem	14.9% (17)	29.7% (44)	23.3% (61)	

Affects social life				Chi-square 4.22
				*p* < 0.05
Not at all a problem	39.6% (44)	33.6% (47)	36.3% (91)	
Minor problem	33.3% (37)	24.3% (34)	28.3% (71)	
Moderate problem	14.4% (16)	22.1% (31)	18.7% (47)	
Serious problem	12.6% (14)	20.0% (28)	16.7% (42)	

Irritates and emotionally affects the individual				Chi-square 7.97
				*p* < 0.01
Not at all a problem	45.6% (52)	32.0% (48)	37.9% (100)	
Minor problem	28.9% (33)	27.3% (41)	28.0% (74)	
Moderate problem	11.4% (13)	15.3% (23)	13.6% (36)	
Serious problem	14.0% (16)	25.3% (38)	20.5% (54)	

Causes sadness or depression				Chi-square 5.99
				*p* < 0.05
Not at all a problem	62.3% (71)	49.0% (70)	54.9% (141)	
Minor problem	18.4% (21)	19.6% (28)	19.1% (49)	
Moderate problem	7.9% (9)	10.5% (15)	9.3% (24)	
Serious problem	11.4% (13)	21.0% (30)	16.7% (43)	

Affects sexual life				Chi-square 11.23
				*p* < 0.01
Not at all a problem	69.0% (78)	49.7% (71)	58.2% (149)	
Minor problem	16.8% (19)	17.5% (25)	17.2% (44)	
Moderate problem	5.3% (6)	16.1% (23)	11.3% (29)	
Serious problem	8.8% (10)	16.8% (24)	13.3% (34)	

Interrupts sleep				Chi-square 6.95
				*p* < 0.01
Not at all a problem	43.1% (50)	30.8% (45)	36.3% (95)	
Minor problem	19.0% (22)	19.2% (28)	19.1% (50)	
Moderate problem	21.6% (25)	18.5% (27)	19.8% (52)	
Serious problem	16.4% (19)	31.5% (46)	24.8% (65)	

**Table 4:** Logistic regression model and factors associated with chronic pain as a function of gender.

Variables	Adjusted RP	CI 95%	*p* value
Pain intensity	1.170	(1.020; 1.342)	0.025
Interference with daily life activities	1.195	(1.038; 1.376)	0.013

Constant	.140		0.000
